# Immune-Related Gene Profile in HIV-Infected Patients with Discordant Immune Response

**DOI:** 10.52547/ibj.3750

**Published:** 2022-10-27

**Authors:** Yeganeh Hamidi, Elaheh Aliasgari, Paria Basimi, Mansour Sajadipour, Kazem Baesi

**Affiliations:** 1 Department of Biology, East Tehran Branch, Islamic Azad University, Tehran, Iran;; 2 Hepatitis and AIDS Department, Pasteur Institute of Iran, Tehran, Iran;; 3 South Health Center, Tehran University of Medical Sciences, Tehran, Iran

**Keywords:** Genes, HIV-1, Immunity, Patients

## Abstract

**Background:**

In spite of many reports on persistent low CD4 T cell counts and change in immune-related gene expression level in patients with HIV infection, there is still uncertainty about significant association between gene expression level and HIV infection in patients with and without DIR. The aim of this study was to compare the expression level of *CD4*, *CCL5*, *IFN-γ*, *STAT1*, *APOBEC3G*, *CD45*, and *ICAM-1* genes in HIV-1-positive patients with and without DIR.

**Methods:**

In this study, 30 HIV-1-positive patients (15 patients with and 15 patients without DIR [control group]) were included. PBMCs of the patients were collected through density radient centrifugation with Ficoll-Hypaque. RNeasy Plus Mini kit was used to extract RNA. Relative expression levels of *CD4*, *CCL5*, *IFN-γ*, *STAT1*, *APOBEC3G*, *CD45*, and *ICAM-1* genes were evaluated by real-time PCR. The data were analyzed using one-way ANOVA.

**Results:**

CD4 T cell counts were significantly lower in DIR patients than the control group (*p* < 0.01). While there was no significant difference in the relative expression levels of *CD4*, *CCL5*, *IFN-γ*, *STAT1*, *CD45*, and *ICAM-1* between patients with DIR and control group, APOBEC3G expression level was significantly higher in the patients with DIR as compare to the control group (*p* < 0.01).

**Conclusion:**

Our findings suggest a significantly higher APOBEC3G expression level in patients with DIR, suggesting the potential role of APOBEC3G in patients with immunological discordance besides its suppressing role in HIV-1 infection. Confirmation of this hypothesis requires further research.

## INTRODUCTION

Research on AIDS/HIV has revealed many aspects of HIV infection. Moreover, morbidity and mortality caused by HIV have significantly been improved by ART. Considering these improvements, AIDS/HIV still continues to be a major public health problem worldwide. Pathogenesis of HIV infection is a complex multifactorial process affected by the virus virulence mechanisms, host immune responses, and genetic factors. PBMCs and immune-related gene expression have been reported to play a major role in immune responses to viral infection, particularly in HIV-infected patients^[^^1^^,^^2^^]^.

In parallel with the CD4 T cell count, VL has also become the most important surrogate marker for HIV infection. The higher the VL, the greater the risk of reduced CD4 T cell counts, leading to the progression or occurrence of AIDS-related illnesses^[^^3^^,^^4^^]^. The total number of VL is a major predictor of the progression of the infection; for instance, the VL decreases in patients taking ART drugs. However, based on observational studies on patients with HIV infection receiving ART, only 40% to 60% of patients significantly reduce VL and increase CD4 cell counts. While HIV-infected patients with concordant positive responses (VL^+^/CD4^+^) have generally favorable outcomes, and those with concordant negative responses (VL^-^/CD4^-^) have much worse outcomes, the prognostic significance of discordant responses (VL^+^/CD4^-^ or VL^-^/CD4^+^) is not still well understood^[^^5^^,^^6^^]^. Notwithstanding experimental and clinical data demonstrating the effectiveness of ART, there is conflicting evidence of immune reconstitution in severely immunocompromised patients, including those with DIR^[^^7^^,^^8^^]^. Some patients, in response to ART, experience DIR, characterized either by a sustained CD4^+^ cell count increase despite persistent viremia or by HIV-1 RNA plasma levels below the limit of detection, which is called immunologic insufficiency. These findings suggest that DIR may be related to the lack of progress in HIV-directed immune responses. DIR is independently associated with an increased risk of mortality^[^^8^^,^^9^^]^. 

Immune-related genes, including *CD4*,* CCL5*, *IFN-γ*,* STAT1*,* APOBEC3G, CD45*, and *ICAM-1*, in PBMCs act a key role in immune responses against HIV-1 infection, and could, hence, be correlated with CD4 T cell depletion in HIV-1 pathogenesis^[^^10^^]^. However, there are few studies focusing on immune-related gene profile in HIV-infected patients experiencing DIR. This is the first study, at least in Iran, compares the expression level of the above-mentioned genes in HIV-infected patients with DIR to that of HIV-infected patients without DIR receiving ART. 

## MATERIALS AND METHODS

This study was conducted on 30 patients with HIV-1-positive infection on regular follow-up at Imam Khomeini Hospital, a reference HIV clinic in Iran (Tehran), during 2017-2019. All the patients received ART for 12 months. Subjects (aged between 18-60 years) were divided into two equal groups (n = 15 in each). The first group included HIV-positive patients (6 women and 9 men) with decreased VL and increased CD4 T lymphocytes, and the second group encompassed HIV-positive patients (3 women and 12 men) with decreased VL and decreased CD4 T cell count (less than 50 cells per mm^3^ in 6-12 months after taking antiretroviral drugs. The inclusion criteria for patients were as follows: receiving ART treatment (emtricitabine, tenofovir, and efavirenz) for at least one year, VL lower than 50 copies/ml for both control and target groups, increase in the number of CD4 cells (25-50 cells per cubic millimeter) during the first year after the start of treatment. In this study, not receiving ART treatment, refusing to complete the consent form, and having a VL above 200 copies/ml were considered as the exclusion criteria. A summary of the clinical characteristics of the study participants is shown in Table 1. 

**RNA extraction**

RNA extraction was performed using RNeasy Plus Mini Kit (QIAGEN GmbH, Germany) based on the instructions provided by the manufacturer. Briefly, 10 ml of whole blood was collected in EDTA Vacutainer tubes. Plasma was isolated from each sample after blood centrifugation and immediately cryopreserved and stored at -70 °C until use. Subsequently, PBMCs were isolated by density gradient centrifugation using Ficoll-Hypaque. Viability of the thawed PBMCs was always >85%. Finally, RNAs were extracted using above kit and stored at -70 °C. The purified RNA was used for RNA-sequencing and quantitative real-time RT-PCR, techniques that require high sensitivity. To reduce external variables affecting the gene expression, transfer and cold chain of blood samples, the number of cells used for mRNA extraction (5 million cells/ml), elution volume, and the One-Step SYBR PrimeScript RT-PCR kit (Takara, Japan) were considered the same for both sample groups.

**Gene expression quantification**

Primers with a concentration of 100 nanomoles were prepared (Table 2). To quantify gene expression, real-time PCR was performed using the One-Step SYBR PrimeScript RT-PCR kit (Takara, Japan). Briefly, *CD4*,* CCL5*,* IFN-γ*,* STAT1*,* APOBEC3G*,* CD45*, and *ICAM-1 *genes were amplified using 3.2 µl of water, 5 µl of One-Step SYBR RT-PCR buffer (2×), 0.2 µl of each primer (10 pM), 0.4 µl of 5 U of enzymes mix 2, and 1 µl of RNA. The real-time PCR experimental run protocol was used as follows: 15 minutes at 42 °C for complementary DNA synthesis, denaturation program at 95 °C for 10 s, 40 times repeated amplification and quantification at 95 °C for 5 s and 60 °C for 45 s, and melting curve program at 60-95 °C with a heating rate of 1 °C per second and a continuous fluorescence measurement. Data were normalized to GAPDH gene, and 2^-ΔCt^ was used to calculate the expression of each gene.

**Table 1:** Individual and clinical characteristics of patients

Characteristics	DIR group	Control group
Male/females (no.)	12/3	9/6
Age/years (mean ± SEM) Mean Std. Dev.	42 ± 9 42.78571 9.158255	39 39 11.68826
		
Risk factor for HIV-1 infection (no.) Homosexual/bisexual Heterosexual Injection drug user Combined	1 9 4 1	1 8 4 2
		
Duration of HIV-1 infection (month) Mean Std. Dev.	43.76 20.78571 6.216338	43.76 20.28571 3.517617
HBV co-infection (no.)	0	0
HCV co-infection (no.)	1	1
PI-containing (no.)	1	1
NNRTI-containing (no.)	14	14
PI and NNRTI containing (no.)	0	0
Viral load	<50-200	<50-200
CD4 trend Mean Std. Dev.	265.99 265.9929 70.37353	516.07 516.0571 282.9624

HIV-1-infected DIR (no. of patients = 15) and control (np15) patients receiving HAART were selected on the basis of having a significant plasma VL (>200 copies/Ml). DIR group and control patients were matched by age, ART, HAART, HBV (hepatitis B virus), HCV (hepatitis C virus), NNRTI, and PI.

**Data analysis**

Data were analyzed using Excel (Microsoft) and GraphPad Prism (Prism 8.0.1). Statistical analysis was performed by applying one-way ANOVA with post hoc Bonferroni test. Comparisons with Gaussian distributions were made using the Kolmogorov-Smirnov test. Data were considered statistically significant if *p* < 0.05. 

## RESULTS

**Fig. 1:**
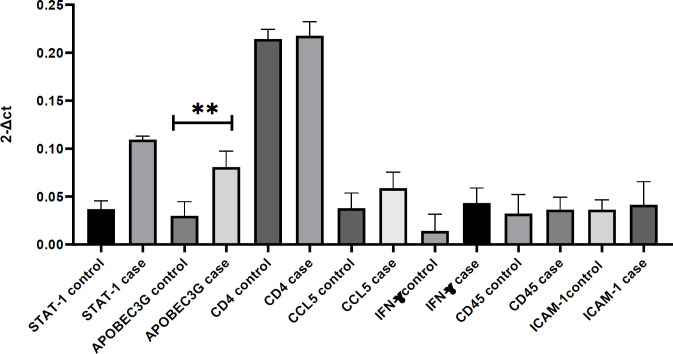
Evaluation of *CD4*,* CCL5*,* IFN-γ*,* STAT1*, *APOBEC3G*,* CD45*, and* ICAM*-gene expression level in PBMCs from HIV-infected patients. *APOBEC3G *gene significantly increases in case group. A 95% CI was considered for all tests (^**^p < 0.01)

In this study, 30 HIV-1-infected patients were divided into DIR (n = 15) and control (n = 15) groups. No statistically significant difference was found between the two groups in terms of age, risk factors for HIV-1 infection, co-infection with hepatitis B or C virus, duration of HIV-1 infection, duration or type of ART, and duration of undetectable VL. However, CD4 T cell counts were significantly lower in the DIR patients than that of the controls. The VL was <50-200 copies/ml in both groups. Also, 14 patients in each group received NNRTI and one received PI. Both groups of patients aged >18 years old were receiving HAART and were HIV-1 seropositive. The average of CD4 T lymphocytes in the DIR and control group was 265.99 and 516.07, respectively (Table 1). The expression level of *APOBEC3G* gene was significantly higher in the DIR than the control group (*p* < 0.01), but the expression of *CD4*, *CCL5*,* IFN-γ*,* STAT1*,* CD45*, and* ICAM-1* genes was insignificant between the two study groups (Fig. 1).

## DISCUSSION

The present study showed no significant difference in the relative expression level of *CD4*,* CCL5*, *IFN- γ*, *STAT1*,* CD45*, and* ICAM-1* genes in PBMCs of the HIV-infected patients receiving ART (control group) and those with DIR receiving ART (DIR group). However, *APOBEC3G* gene expression level was higher in the DIR group than the control patients. 

Previous studies have disclosed that drug strategies aimed at stabilizing *APOBEC3G* in HIV-infected cells are required to be investigated as potential HIV/AIDS treatments. Although certain experimental and clinical surveys have demonstrated that the members of the APOBEC family of cytidine deaminases can suppress HIV-1 infection, it has not been clearly determined that how they exert their antiviral effects^[^^11^^,^^12^^]^. *APOBEC3G* expression level in PBMCs has been reported to be correlated with VLs or CD4 T cell counts during primary infection. However, there is an insufficient data on the interplay between HIV-1 and *APOBEC3G* expression *in vivo*, especially during primary infection when rapid viral replication occurs, followed by resolution of viremia and establishment of steady state equilibrium between the virus and the body immune responses^[^^13^^]^. Few reports have indicated that the expression level of *APOBEC3G* is correlated directly with CD4^+^ cell count, as well as T CD4^+^ cell ratio^[^^14^^]^. Our findings displayed that the increased expression level of *APOBEC3G* gene may be correlated with CD4 cell count, apart from the function of this gene in the reduction of VL. Previous studies have reported the effective potential role of *APOBEC3G* gene expression in the inhibition of viral replication in HIV-1-infected patients and introduced this gene as a biomarker for immunotherapy^[^^15^^,^^16^^]^.

**Table 2:** Sequences and the product length of primer used in this study

**Gene**	**Primer**	**Product length (bp)**
*APOBEC3G*	F: GAGGAAACACAGTGGAGCGAATG R: AGCACAGCCAGACGGTATTC	96
		
*CCL5*	F: GCAGTCGTCCACAGGTCAAG R: TCTTCTCTGGGTTGGCACAC	137
		
*CD4*	F: GGAGGCAAAGGTCTCGAAGC R: GGGCAGAACCTTGATGTTGG	124
		
*CD45*	F: GACCCCTTACCTACTCACACC R: GAAGGTGAGGCGTCTGTACTG	187
		
*ICAM-1*	F: CGGAAGGTGTATGAACTGAGCAA R: AGGAAGGTTTTAGCTGTTGACTG	95
		
*IFN-γ*	F: AGTGATGGCTGAACTGTCGC R: CTGGGATGCTCTTCGACCTC	109
		
*STAT1*	F: GGTGGCAGGATGTCTCAGTGG R: GCTCCCAGTCTTGCTTTTCTAACC	142
		
*GAPDH*	F: TCGTGGAAGGACTCATGACC R: CCATCACGCCACAGTTTCCC	88

According to the results of this study, HIV-1 patients who had received ART exhibited a higher level of immunity than those who had not received ART. Research has shown that long-term nonprogressors spontaneously regulate HIV-1 replication and transcription through the TGF-β signaling pathway and help maintain the normal range of CD4^+^ T cells^[^^17^^,^^18^^]^. Recent studies have suggested that the expression level of *CD45* is a critical parameter for T-cell development, and *CD45* has a crucial role in apoptosis induced by HIV-1 gp120, as a key part of the T-cell receptor pathway^[^^19^^,^^20^^]^. Indeed, *CD45* causes a significant increase in T-cell proliferation and cytokine production; hence, the *CD45* expression level is a critical parameter for T-cell development. However, in our study, the expression level of *CD45* and its count did not show any significant change in the patients. Further research is required to determine “how” and “why” the *CD45* expression level has not significantly changed in the patients.

While different reports on HIV infection have revealed the effect of enhanced *STAT1* activation on CD4 T cell activation and function^[^^21^^,^^22^^]^, we did not observe any significant difference of *STAT1* gene expression level between the control and DIR patients receiving ART. Our findings also indicated no significant difference in *IFN-γ* expression level between the two groups of patients. In line with this finding, it has been shown that *IFN-γ* has no direct antiviral activity against HIV-1 infection in primary cultures, as supported by the *in vivo* findings of *IFN-γ* therapy in infected subjects^[^^10^^,^^23^^]^. In contrast, certain studies have reported that this cytokine can increase the cytotoxic T lymphocytes and natural killer cell activities against HIV-1-infected cells. In addition, *IFN-γ* upregulates the MHC-II pathway supporting the antigen-specific activation of CD4^+^ T cells^[^^24^^-^^26^^]^. Research has exhibited that in primary HIV infection, *ICAM-1* significantly increases and is involved in promoting inflammatory responses against HIV infection. However, few studies have indicated the major role of *ICAM-1* in enhancing CD4 cells^[^^27^^]^. We also did not find any association between *ICAM-1* expression level and CD4 cells count in our study. 

*CCL5/RANTES*, a natural ligand of *CCR5*, is one of the main suppressors of HIV. Among various chemokines, *CCL5* has been introduced as a regulator for activation and expression of normal T cells^[^^28^^]^. Although the role played by *CCL5* in the treatment of HIV/AIDS has recently been reported^[^^29^^]^, our study found no significant change in the *CCL5* expression level in patients receiving ART. A recent study has suggested that alterations in CD4 cell concentrations may control HIV-1 infections and disease progression. CD4 is also a key target for *Nef* protein. The HIV-1 *Nef* protein is a critical factor in viral pathogenesis^[^^30^^]^. Therefore, the viral *Nef* protein may have a direct connection with CD4 degradation. However, our findings disclosed that the VL reached undetectable level, but CD4 cells did not significantly increase in the DIR group. 

Several methodological limitations should be considered when interpreting the results of the present study. The main downside is the small sample size, which restricts the precision of estimates and power for detecting differences between the study groups. However, we explored that HIV-1 patients receiving ART with high *APOBEC3G* expression levels had low VL, despite having a lower proportion of CD4^+^ T cells than normal individuals. Actually, HIV encodes a protein called *Vif* that binds to *hA3G* and destroys the antiviral effects of *APOBEC3G*, but such bindings vary depending on the mutations occur in the *Vif* gene of HIV-1. In the absence of the *Vif* protein, *APOBEC3G* surrounds the virus and restricts viral replication through an independent deaminase mechanism^[^^31^^,^^32^^]^. Contrary to our expectation, the increased expression level of *APOBEC3G* gene was not followed by a significant elevation in the number of CD4T cells in DIR patients. In our study, different expressions of *APOBEC3G* and its correlation with DIR suggest that this gene likely has no important function in elevating the CD4 T cells count in HIV infection. However, no significant difference was observed in the relative expression level of *CD4*,* CCL5*, *IFN-γ*,* STAT1*,* CD45*, and* ICAM-1* genes between the two patient groups. 

Overall, the result of this study indicate a significantly higher *APOBEC3G* expression level in patients with DIR, denoting that this gene may be responsible for immunological discordance in patients, in addition to its suppressing role in HIV-1 infection. More investigation is needed to confirm this hypothesis in future.

## DECLARATIONS

### Acknowledgments

The authors would like to acknowledge all participants who participated in this study. 

### Ethical statement

The above-mentioned sampling protocols were approved by the Ethics Committee on Biomedical Research in the Pasteur Institute of Iran, Tehran (ethical code: IR.PII.REC.1396.49). Informed consent was obtained from each participant included in the study.

### Data availability

The analyzed data sets generated during the study are available from the corresponding author on reasonable request.

### Author contributions

YH: carried out research plan; EA, PB, MS, and KB suggested the idea of research and supervised all practical steps. All authors shared in writing, statistical analysis, and revision process, and final approval.

### Conflict of interest

None declared.

### Funding/support

This study (grant number 1016) was funded by the Pasteur Institute of Iran, Tehran and Islamic Azad University, East Tehran Branch, Iran. 
